# Gait Pattern in People with Multiple Sclerosis: A Systematic Review

**DOI:** 10.3390/diagnostics11040584

**Published:** 2021-03-24

**Authors:** María Coca-Tapia, Alicia Cuesta-Gómez, Francisco Molina-Rueda, María Carratalá-Tejada

**Affiliations:** 1 Masters School, Rey Juan Carlos University, 28933 Madrid, Spain; macocata12@gmail.com; 2 Motion Analysis, Ergonomics, Biomechanics and Motor Control Laboratory (LAMBECOM), Department of Physical Therapy, Occupational Therapy, Rehabilitation and Physical Medicine, Faculty of Health Sciences, Universidad Rey Juan Carlos, Alcorcón, 28922 Madrid, Spain; francisco.molina@urjc.es (F.M.-R.); maria.carratala@urjc.es (M.C.-T.)

**Keywords:** multiple sclerosis, gait, biomechanics, three-dimensional motion capture systems

## Abstract

The aim of the present systematic review was to describe the gait pattern in people with multiple sclerosis (MS) by compiling the main findings obtained from studies using three-dimensional capture systems of human movement. The search was carried out in PubMed, Web of Science, Physiotherapy Evidence Database (PEDro), and the Cumulative Index to Nursing and Allied Health (CINAHL) databases. Studies that used three-dimensional gait analysis systems and that analyzed spatiotemporal, kinematic, kinetic, or electromyographic parameters, were included. The quality of the studies was assessed using the Critical Review Form–Quantitative Studies scale. 12 articles were included with 523 (342 women and 181 men) people with a diagnosis of MS. The present work suggests that people with MS have a decrease in speed and stride length, as well as an increase in double-stance intervals during gait. Likewise, it is common to observe a decrease in hip extension during the stance period, a decrease in knee flexion in the swing period, a decrease in ankle dorsiflexion in the initial contact and a decrease in ankle plantar flexion during the pre-swing phase. The subjects with MS decrease the hip extensor moment and the ankle power during the stance period of walking.

## 1. Introduction

Multiple sclerosis (MS) is a chronic, degenerative, and demyelinating disease, and one of the primary causes of disability in young adults in Europe and North America [1,2,3,4,5]. MS can cause sensory (40%), pyramidal (40%), cerebellar (25%), and visual symptoms (20%) [6,7]. Gait deficiency is one of the main causes of disability in people with MS, and from the perspective of the patients it is the most challenging symptom [8,9,10]. In this sense, several authors have estimated that 15 years after the diagnosis of the disease, half of the patients will require assistance to walk and 10% will use a wheelchair [11,12].

Gait abnormalities after a disorder of the central nervous system (CNS) are quite different, although it has been observed that some of the most common findings are a decrease in step length and single support time, slower speed, increase in step width, and decrease in ankle dorsiflexion angle and propulsive force [13,14]. In this sense, a typical gait pattern has not been described in MS unlike in other neurological disorders such as Parkinson’s disease or stroke.

Various studies have described several abnormalities in the gait pattern of people with MS. Benedetti et al. showed a reduced speed of progression, shorter strides, and prolonged double support intervals in minimally impaired MS patients [15]. Preiningerova et al. observed that gait velocity decreases with increasing expanded disability status scale levels (EDSS) [16]. Despite these findings, walking abnormalities in MS patients are, nevertheless, poorly characterized.

For gait assessment, a three-dimensional computerized analysis may be considered the criterion standard because it provides objective data on electromyography as well as kinetic and spatiotemporal parameters [1]. These tools assess the coordination of spatial and temporal aspects of the gait pattern.

As impaired gait coordination and recovery are of prime significance in neurorehabilitation, we conducted a systematic review to evaluate the gait abnormalities in people with MS registered though studies that used three-dimensional motion capture systems.

## 2. Materials and Methods

### 2.1. Search Strategy

The search was conducted from April to May 2020 using PubMed, Web of Science, Physiotherapy Evidence Database (PEDro), the Cumulative Index to Nursing and Allied Health, Web of Science, and the Cochrane Library. Only cross-sectional articles published in English, French, or Spanish were selected, without limits on the year of publication. The combinations of keywords used were as follows: (“Visual” OR “observational” OR “pattern”) AND (“Gait” OR “ambulation” OR “walk”) AND (“Assessment” OR “evaluation” OR “Test” OR “scale” OR “measure” OR “tool” OR “analysis” OR “profiling”) AND “Multiple Sclerosis.”

### 2.2. Study Selection

Studies that met the following inclusion criteria were included: (1) cross-sectional studies that use three-dimensional analysis to assess walking and evaluate the spatiotemporal, kinematic, kinetic, or electromyographic parameters; (2) the population under study is composed of adult patients with MS (aged ≥ 18y). This systematic review excluded articles according to the following exclusion criteria: (1) studies that only use observational gait assessment scales; (2) studies that assess aspects of gait that are different from coordination of the gait pattern, such as walking resistance or level of walking impairment; studies without control group.

### 2.3. Data Collection

The general characteristics of the studies, including number of patients, type of MS, 3-dimensional motion capture system used, and biomechanical parameters examined, were extracted.

Two of the authors (F.M. and M.C.) conducted 2 independent assessments of the abstracts obtained from the electronic search. The reviewers decided which articles could potentially meet the inclusion criteria, and the works selected by each author were compared. Any disagreements were resolved after discussions with an independent third reviewer. For the studies that met the inclusion criteria, the full text articles were obtained.

### 2.4. Methodological Quality

The quality of the studies was assessed using the Critical Review Form–Quantitative Studies scale. This assessment tool assesses the rigor and bias of the method using a combination of dichotomous (yes/no) and descriptive elements. It incorporates 8 items, and each item is scored with 0 (does not meet the criteria) or 1 (meets the criteria). A higher score implies greater methodological quality of the evaluated study [17]. Finally, this review followed the PRISMA guidelines [18].

## 3. Results

### 3.1. Description of the Studies

**Figure 1:**
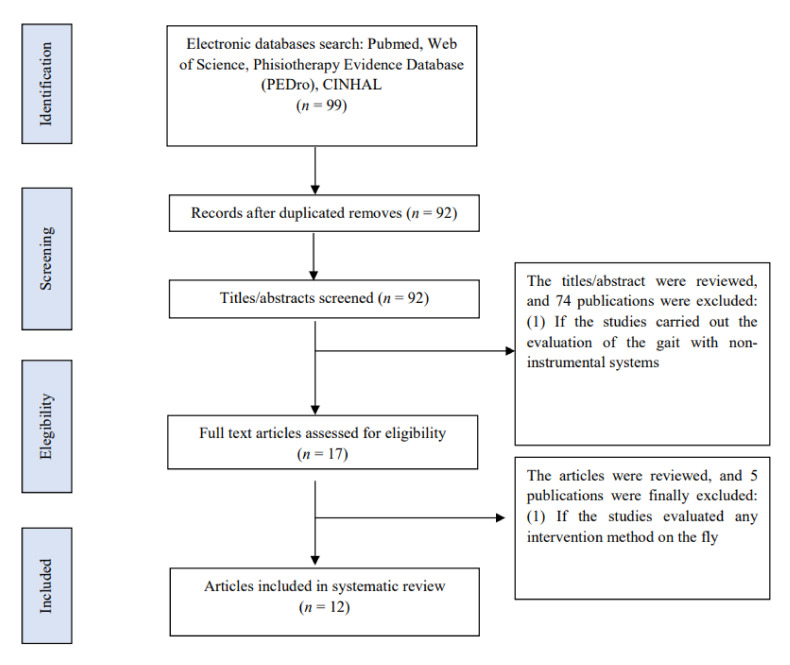
Flow chart.

Ninety-nine articles were found in the literature search. Once the abstracts were read, 17 of them were selected for further review and critical reading. Additionally, the references of these articles were reviewed to avoid loss of information that was not found in the bibliographic search, without obtaining any results. Finally, 5 articles were excluded in this review [19,20,21,22,23] and 12 were included [24,25,26,27,28,29,30,31,32,33,34,35] (Figure 1).

All included studies enrolled 523 (342 women and 181 men) people with MS. Only three publications included the three types of MS: remitting recurrent (RR), progressive primary (PP), and progressive secondary (SP) [24,25,27]. In four studies, the authors only included RR-type MS [26,28,29,32] and two combinations of two types [33,34], and the remaining three did not specify what type of MS their participants had diagnosed [30,31,35]. All studies except one (which used the Hauser Index to classify participants as mild or moderately disabled) [34] classified the intervention subjects with the EDSS: two of them included participants with mild to moderate disability (EDSS < 4) [26,29], three included participants with moderate disability without aids in ambulation (EDSS < 5.5) [24,28,35], and the remaining six included users with mild to moderate disability with ambulation assistance using an assistive product (EDSS < 6.5) [25,27,30,31,32,33]. All studies except one [30] evaluated the spatiotemporal parameters of gait in MS, six included kinematic parameters [24,25,26,28,30,34], two included kinetic parameters [30,34], and two studies evaluated muscle activity during gait [28,34] (Table 1).

### 3.2. Summary of the Results

All the authors found significant differences in the spatiotemporal parameters, except one, which had a sample of MS users with minimal disability [26]. Most studies showed a decrease in gait speed and stride and step length [25,29,31,32,33,34,35]. Severini et al. [25] showed differences in gait speed according to the level of disability (low disability: 97.5 cm/s; moderate disability: 52 cm/s; severe disability: 23.9 cm/s vs. control: 139.2 cm/s). Remilius et al. [27] reported a decrease in the swing period duration during walking in MS patients (MS: 230 mm vs. control 253 mm) while Pau et al. [28] expressed an increase in the percentage of the stance period (MS: 65.48–67.75% vs. control: 61.79–61.32%). Two studies [31,32] found an increase in the step width (Socie et al.: MS: 12.6 cm vs. control 8.6 cm; Kalron et al.: MS:13.8 cm vs. control: 11.0 cm).

Regarding the kinematic parameters, two studies recorded data from the pelvis. Severini et al. [25] found an increase in pelvic tilt and hiking throughout the gait cycle. Morel et al. [26] observed a reduced pelvis tilt and obliquity during walking in MS patients with EDSS ≤ 2.

At the hip joint, the studies described a decrease in the maximum extension in the stance period in patients with mild to moderate involvement (EDSS ≤ 6.5) (moderate disability: −5.5°, high disability: 1.4° vs. control: −12.50°) [25]. However, in patients with less involvement (EDSS < 4), the studies did not suggest significant differences in the hip pattern [29].

Regarding the knee joint, publications described a decrease in the maximum flexion achieved during the swing period [24,25] (Filli et al.: MS: 35° vs. control 55°; Severini et al.: low disability: 42.2°, moderate disability: 30.6°; severe disability: 14.2° vs. control: 57.0°). At the ankle joint, several studies observed a decreased dorsiflexion during the stance [24,25,30] (Filli et al.: MS: −10° vs. control: −5°; MS: −10° vs. control: 0°; Severini et al.: severe disability: 5° vs. control: 12°), and a decreased plantar flexion in the pre-swing phase [26,30] (Morel at al.: MS: −14.67° vs. control: −20.0°) (Table 2).

The kinetic characteristics are described in two publications [30,34]. Huisinga et al. [30] and Kelleher et al. [34] analyzed the joint internal moments. Both authors observed a decrease in the hip extensor moment (Huisinga et al.: MS: 0.650 Nm/Kg vs. control: 0.789 Nm/Kg; Kelleher et al.: EM: 0.86 Nm/Kg vs. control: 0.99 Nm/Kg) during the mid-stance phase. In addition, Huisinga et al. [30] registered a decrease in the ankle power generated (MS: 2.440 W/kg vs. control: 3.121 W/Kg).

The electromyographic characteristics of the gait pattern were only evaluated in two studies. Pau et al. [28] described an increase in rectus femoris activation throughout the gait cycle in individuals with spasticity, while Kelleher et al. [34] found an increase in the percentage of activation of the plantar flexor muscles during the stance period.

### 3.3. Methodological Quality

The methodological quality of the included studies is detailed in Table 3. The scores obtained range from 11 [25,28] to a maximum of 15 [24]. All studies reflect the research purpose and the design of the protocol. The sample is well described in all studies, but only two works justified the sample size [24,33], which increases the probability of the appearance of selection bias.

## 4. Discussion

The gait pattern in people with MS depends on the location of the injuries and the degree of damage. For these reasons, it is difficult to homogenize the gait abnormalities and stablish a standardized atypical pattern. All the studies included in this review have reported involvement of the spatiotemporal parameters of the gait, specifically a decrease in the speed and stride and step length.

Regarding the kinematic and kinetic parameters, those with MS walk with a reduced hip extension and hip extension moment in the stance period [25,30,34]. This pattern could be explained by an increase in the quadricep tone in patients with spasticity or by a weakness of the hip extensor muscles. The weakness of the hip extensor musculature could explain the reduction in hip extensor moment described by several articles during the stance period [25,34]. The restriction of hip extension is correlated with a higher risk of falls in people with neurological pathology [36] and in elderly people [37,38].

At the knee joint, there is some consensus that people with MS reduce the flexion during the swing period [24,25,28,34]. Some authors also observed an increase in knee flexion in the loading response [34]. Therefore, impaired knee flexion could be one of the main kinematic features of MS gait [24]. This is congruent with previous studies reporting that impaired knee flexion is an important MS-associated gait characteristic and a valid predictor of walking function/gait speed in these patients [24,39]. The reduced knee flexion during the swing phase may be caused by paresis (e.g., hamstrings), increase in muscle tone (e.g., rectus femoris) or decreased push-of power in the ankle joint (e.g., gastrocnemius) [24,40].

Regarding the ankle joint, the main alteration described is the decreased dorsiflexion during the stance and the restricted plantar flexion during the toe off [24,25,26,30]. These findings could be related to an increase in the plantar flexor muscles tone during the stance period in subjects with spasticity [30]. This pattern is congruent with the kinetics recorded in two of the studies, which report a decrease in the power generated in the pre-swing phase [30]. The ankle power, which is the amount of power (energy) transmitted through the action of forces generated by the musculotendinous structures, is considered a good predictor of the impairment of stride length in elderly people and is positively correlated with stride length and speed [41].

The restriction of motion at hip, knee and ankle could cause an increase in the pelvic tilt and hiking during walking, as reported Severini et al. [25]. However, this pelvis pattern has not been observed in those with MS with mild disability [26]. Therefore, the pelvic pattern could be affected by disease progression and as compensation for restricted movement in the hip, knee, or ankle joints during walking.

These alterations observed in the kinematic pattern of subjects with MS should be considered in the evaluation process, since this asymmetrical pattern demonstrates the gait deterioration throughout the course of the disease. Rehabilitation should prevent asymmetries such as decreased hip extension or knee flexion during walking, as these are findings that indicate deterioration or progression of the disease. Several authors have demonstrated that rehabilitation improves walking in people with MS. Tramonti et al. [42] showed that an intensive task-oriented circuit training improved fatigue, perceived ambulatory function and knee force in mildly impaired MS subjects. Benito-Villalvilla et al. [43], in a systematic review, expressed that motor imagery and its combination with relaxation techniques improve fatigue, gait, balance, depression and quality of life in people with MS. In addition, Robinson et al. [44] showed that treadmill training may be an effective form of task-specific training for improving walking in people with MS.

The gait described in this review could be greater in MS patients with spasticity-related signs. They present a greater reduction in speed and higher increase in the percentage double support periods. On the other hand, the main gait characteristics in MS patients with cerebellar involvement are an increase in the step width [31,32]. This is congruent with other works which show a more asymmetric gait pattern in MS patients with spasticity-related signs compared to the those with cerebellar involvement [45,46]. There are also other publications that have compared the risk of falls in both groups, and there does not seem to be conclusive data on this [47,48,49,50]. These studies reported the possibility of falling is mainly correlated with the level of disability and the presence of visual impairment.

Regarding the EDSS levels, one of the studies carried out with patients with mild disability (EDSS ≤ 2) did not find significant differences with respect to the control group [26]. Another study [25], which divided the participants into mild, moderate, and severe disability, did find an impairment of speed and stride length in the mild group. The rest of the studies had participants with moderate disabilities, and although they show that the parameters increase their asymmetry with the progression of the disease, they do not specify in which EDSS score it occurs. This could suggest that the gait pattern alterations do not appear in the earliest phase of the disease and that they tend to worsen as the disease progresses.

This review has some limitations that should be highlighted. First, only papers in English, Spanish or French were considered, so there may be works that have not been studied. Second, some of the articles included have small samples, the years of evolution of the disease fluctuate, and the data collection systems are different, which contributes heterogeneity to the results described. Third, this review cannot draw conclusions about differences in gait pattern according to several clinical variables that have not been considered in most of the included studies (i.e., numbers of clinical relapses in patients with RR-MS; site of demyelinating lesions) In addition, this review considered articles that report the MS clinical phenotype, but they did not establish comparisons between gait abnormalities pattern and MS phenotype. In addition, there are three articles that did not report the MS clinical phenotype. Further research on these comparisons should be considered.

## 5. Conclusions

The present review suggests that people with MS have a decrease in speed and stride and step length and an increase in the step width. It is common to observe a decrease in hip extension during the stance period, a decrease in knee flexion in the swing period, a decrease in ankle dorsiflexion in the initial contact and a decrease in ankle plantar flexion during the pre-swing phase. The subjects with MS walk with a decreased hip extensor moment and ankle power.

Regarding to the gait pattern in different MS subgroups, it seems that walking is worse in patients with spasticity-related signs compared to those with cerebellar signs. All these parameters appear to worsen as the EDSS score progresses and are not seen in mildly disabled individuals. These results may help to improve rehabilitative gait training in MS patients and to develop specific exercise programs. Furthermore, these findings are relevant for future research to compare the gait pattern in patients with different types of multiple sclerosis.

## Figures and Tables

**Table 1:** Participant characteristics of the included articles.

Study	Multiple Sclerosis Participants					Control Participants		
	N	Sex (F:M)	Age	Type	EDSS	N	Sex (F:M)	Age
Filli et al. 2018 [24]	37	24:13	48.6	22 RR 2 PP 13 SP	1–4.5	20	12:8	48.8
Severini et al. 2017 [25]	51	30:21	51	7 RR 18 PP 26 SP	2–6.5	10	2:8	36.7
Morel et al. 2017 [26]	34	22:12	36.32	34 RR	≤2	22	16:6	36.8
Remelius et al. 2015 [27]	16	16:0	51.3	15 RR 2 PP 2 SP	2.5–6	19	19:0	51.8
Pau et al. 2015 [28]	19	12:7	54.6	19 RR	2.5–4.5	19	7:12	47.1
Kalron et al. 2014 [29]	20	8:12	36.3	20 RR	<4	20	10:10	34.3
Huisinga et al. 2013 [30]	31	26:5	46.2	NE	0–6.5	31	23:8	42
Socie et al. 2013 [31]	86	73:13	52.4	NE	2.5–6.5	20	16:4	50.9
Kalron et al. 2013 [32]	87	50:37	40.9	87 RR	<6	25	14:11	38.5
Gianfrancesco et al. 2011 [33]	11	7:4	53	8 RR 3PP	3.5–6	13	6:7	47
Kelleher et al. 2010 [34]	16	8:8	42.13	NE RR NE SP	–	10	5:5	37.2
Givon et al. 2009 [35]	81	53:28	36.2	NE	≤5.5	25	17:8	34.2

N: number of participants; EDSS: expanded disability status scale; F: female; M: male; remitting recurrent MS (RR); progressive secondary MS (SP); progressive primary MS (PP); no specified (NE).

**Table 2:** Synthesis of the results.

Study	Outcome Measures	Gait System Used	Main Results
Filli et al. 2018 [24]	Spatiotemporal parameters Kinematic Kinetic	Vicon Motion System^®^	Decrease in speed (MS: 6.2 s vs. Control: 3.6 s; *p* < 0.0001). Kinematics: decreased knee flexion during swing phase (MS: 35° vs. control 55°; *p* <0.05) and decreased ankle dorsiflexion during the initial contact and swing period (MS: −10° vs. control: −5°; MS: −10° vs. control: 0°; p < 0.05). Follow-up after one year: greater deterioration in gait of the spastic-paretic type (*p* = 0.0473) and greater deterioration in knee parameters (*p* = 0.0129).
Severini et al. 2017 [25]	Spatiotemporal parameters Kinematic	Vicon Motion System^®^	Differences in spatiotemporal parameters according to the degree of MS disability: decrease in speed (low disability: 97.5 cm/s; moderate disability: 52 cm/s; severe disability: 23.9 cm/s vs. control: 139.2 cm/s; *p* < 0.001). Stride length decreased (low disability: 111.9 cm; moderate disability: 82.6 cm; severe disability: 55.8 cm vs. control: 139.2 cm; *p* < 0.001). Pelvis: increased pelvic tilt and hiking. Hip Kinematic: decreased extension during pre-swing (moderate disability: −5.5°, high disability: 1.4° vs. control: −12.50°; *p* < 0.0001); knee: decreased flexion during swing period (low disability: 42.2°, moderate disability: 30.6°; severe disability: 14.2° vs. control: 57.0°; *p* < 0.0005); ankle: decreased dorsiflexion during stance period (severe disability: 5° vs. control: 12°; *p* = 0.01).
Morel et al. 2017 [26]	Spatiotemporal parameters Kinematic	Vicon Motion System^®^	Pelvis Kinematic: decreased pelvis tilt (MS: 9.09° vs. control: 11.08°; *p* = 0.033) and reduced obliquity range (MS: 10.33° vs. control: 12.83°, *p* < 0.001). Ankle: increased dorsiflexion during mid-stance and decreased plantar flexion in pre-swing (MS: −14.67° vs. control: −20.0° *p* < 0.001).
Remelius et al. 2015 [27]	Spatiotemporal parameters Kinematic Kinetic	Oqus System^®^	Duration of the swing period is decreased (MS: 385 ms vs. control: 401 ms; *p* = 0.004). Anteriorized COM in the swing period (MS: 230 mm vs. control 253 mm; *p* < 0.001).
Pau et al. 2015 [28]	Spatiotemporal parameters Kinematic Electromyography	Smart-D System	Decrease in speed (MS: 0.42 m/s vs. control: 1.12 m/s; *p* < 0.001) and increase in the percentage of the stance period (MS: 65.48–67.75% vs. control: 61.79–61.32%; *p* = 0.021). Increase in the rectus femoris activation throughout the gait cycle (RMS in MS: 0.644–0.639 W/s vs. control: 0.504–0.493 W/s; *p* < 0.001).
Kalron et al. 2014 [29]	Spatiotemporal parameters Kinetic	Zebris FDM-T^®^	Decrease in self-paced gait (MS: 3.24 km/h vs. control: 3.52 km/h; *p* = 0.32), cadence 99.0 step/min vs. control 100.4 step/min; *p* = 0.75), step width (MS: 14.1 cm vs. control: 10.9 cm; *p* = 0.02).
Huisinga et al. 2013 [30]	Spatiotemporal parameters Kinetic	EvaRT 5.0, Motion Analysis^®^	Ankle Kinematic: increased plantar flexion during loading response (MS: 5.47° vs. control: 6.58°; *p* = 0.016) and decreased during pre-swing (MS: 13.53° vs. control: 16.88°; *p* = 0.022). Decreased hip extensor torque during early stance. (MS: 0.650 Nm/Kg vs. control: 0.789 N·m/Kg; *p* = 0.157). Decreased ankle power absorption in early stance. (Ankle MS: −0.398 W/kg vs. control: −0.601 W/Kg; *p* = 0.015. Knee: −0.675 W/Kg vs. control: −1.021 W/Kg; *p* = 0.006). Decreased power generation in late stance (MS: 2.440 W/kg vs. control: 3.121 W/Kg; *p* = 0.008).
Socie et al. 2013 [31]	Spatiotemporal parameters	GAITRite TM^®^	Decrease in speed (MS: 1.0 m/s vs. control: 1.4 m/s; *p* < 0.001) and step length (MS: 58.0 cm vs. control: 73.8 cm; *p* < 0.001). Increased step width (MS: 12.6 cm vs. control 8.6 cm; *p* = 0.002) and extended step time (MS: 603 ms. vs. control: 530 ms; *p* < 0.001).
Kalron et al. 2013 [32]	Spatiotemporal parameters	Zebris FDM-T^®^	Decrease in speed (MS: 2.2 Km/h vs. control: 3.5 km/h; *p* < 0.001) and increase in step width (MS:13.8 cm vs. control: 11.0 cm; *p* < 0.001).
Gianfrancesco et al. 2011 [33]	Spatiotemporal parameters	GAITRite TM^®^	Decrease in speed (MS: 73.3 cm/s vs. control: 134.4 cm/s; *p* < 0.10) and stride length (MS: 103.7 cm vs. control: 144.3 cm; *p* < 0.10).
Kelleher et al. 2010 [34]	Spatiotemporal parameters Kinematic Kinetic Electromyography	Vicon Motion System^®^	Alteration in all spatiotemporal parameters except cadence (Speed MS: 1.2 m/s vs. control: 1.42 m/s. Step length (MS: 0.63 m vs. control: 0.80 m; *p* < 0.05). Decrease in external hip moment (EM: 0.86 Nm/Kg vs. control: 0.99 Nm/Kg; *p* < 0.05). Maximum propulsive anteroposterior force was reduced during pre-swing (MS: 1.65 N/Kg vs. control: 2.42 N/kg; *p* < 0.05). Increase in the percentage of activation of the gastrocnemius during the gait cycle (MS medial gastrocnemius: 29.5% vs. control: 11%; MS Lateral gastrocnemius: 28.82% vs. control: 14.27%; *p* < 0.05).
Givon et al. 2009 [35]	Spatiotemporal parameters	GAITRite TM^®^	Decrease in speed (MS: 85.5 cm/s vs. control: 138.6 cm/s; *p* < 0.001), cadence (MS 94.4 p/min vs. control: 115.2; *p* < 0.001) and step length (MS: 45.3 cm vs. control: 72.1 cm; *p* < 0.001).

COM: center of mass; MS: multiple sclerosis; Units: s: seconds; o: degrees; cm/s: centimeters/second; cm: centimeter; ms: millisecond; mm: millimeter; m/s: meter/second; %: percentage; Km/h: kilometer/hour; mm2: square millimeter; Nm/Kg: newton-meter/kilogram; W/Kg: watt/kilogram (power); p/s: step/second.

**Table 3:** Methodological quality of the included articles.

ITEMS	1	2	3	4	5	6	7	8	9	10	11	12	13	14	15	TOTAL
Filli et al. 2018 [24]	1	1	1	1	1	1	1	1	1	1	1	1	1	1	1	15
Severini et al. 2017 [25]	1	1	1	1	0	1	1	1	1	1	1	0	0	0	1	11
Morel et al. 2017 [26]	1	1	1	1	0	1	1	1	1	1	1	1	0	0	1	12
Remelius et al. 2015 [27]	1	1	1	1	0	1	1	1	1	1	1	1	0	0	1	12
Pau et al. 2015 [28]	1	1	1	1	0	1	1	1	1	1	1	0	0	0	1	11
Kalron et al. 2014 [29]	1	1	1	1	1	1	1	1	1	1	1	0	0	0	1	12
Huisinga et al. 2013 [30]	1	1	1	1	0	1	1	1	1	1	1	1	0	0	1	12
Socie et al. 2013 [31]	1	1	1	1	0	1	1	1	1	1	1	1	0	0	1	12
Kalron et al. 2013 [32]	1	1	1	1	0	1	1	1	1	1	1	1	1	0	1	13
Gianfrancesco et al. 2011 [33]	1	1	1	1	1	1	1	1	1	1	1	1	0	0	1	13
Kelleher et al. 2010 [34]	1	1	1	1	0	1	1	1	1	1	1	1	0	0	1	12
Givon et al. 2009 [35]	1	1	1	1	1	1	1	1	1	1	1	1	0	0	1	13

Items: 1. study purpose; 2. literature; 3. design; 4. sample described; 5. sample size justified; 6. outcome measures reliable; 7. outcome measures valid; 8. description of the intervention; 9. contamination; 10. cointervention; 11. statistical significance of the results; 12. analysis method(s); 13. clinical importance; 14. dropouts; 15. conclusions.
